# Supplementary material for: Prevalence and factors associated with female genital mutilation/cutting among Tanzanian women who gave birth in the five years prior to the survey: A population-based study
Source: PLoS One. 2024 Dec 30;19(12):e0310337. doi: 10.1371/journal.pone.0310337 (PMC11684563; doi:10.1371/journal.pone.0310337)
Supplement: Attachment — Submitted filename: PLOS ONE.docx [file pone.0310337.s002.docx]

1**. The study presents the results of original research**.

It seems a review as it has critically evaluated the available data. It will help for a quick reference otherwise; the full version of the DHS has been available online for anybody.

The data has been extracted from the DHS. My concern here is the data has been collected from permanently residents and visitors who stayed for one night, is it really make sense to collect a data from visitors especially on sensitive and culture related topics? Such kinds of practices are done secretly, how visitors will have the information?

The other concern is In Tanzania the Government is working a lot to create awareness on FGM/C which has been a cause for the dropping of FGM/C from 10% (DHS 2015-16) to 8% (DHS 2022) as it has been justified in the report, there is a discrepancy between the main report and this research. The other point is this is purely cultural issue not related to knowledge and religion not only for Tanzania but also all over Africa. So such kinds of sensitive issue it will be difficult to change the context only be awareness creation and the approach for the research should be different as it has to use behavioral and norm related model and theories to know the unwritten and unspoken hidden norms related with FGM/C, this will help all concerned bodies to critically plan to challenge the barriers. I do recommend PLOS ONE to critically evaluate papers for a real and meaningful contribution in research world, not for publication purpose but it has to be used as an input for the decision makers and other stake holders.

From my knowledge is concerned, in Africa the problem is no one is interested to explore a new knowledge but duplication. PLOSE ONE is a appreciated journal which challenges researchers than others, so avoiding such kinds of researches have to be avoided. There are lots of issues should be answered or little is known, the answer of this research topic is known “we have to avoid duplication”.

**2. Results reported have not been published elsewhere.**

The most recent DHS result has been published; it is enough for reference and input purpose. Policy makers and other concerned bodies can refer the officially launched DHS report. For such kinds of culturally sensitive areas, we should use another innovative method to understand the deep rooted causes to make a difference. For the publication purpose there are other journals open for any kinds of papers.

**3. Experiments, statistics, and other analyses are performed to a high technical standard and are described in sufficient detail**.

The result part is the main part of the paper which peoples quickly refer to understand the context but in this paper it has been written mainly in numbers (not interpreted in words) which can cause the readers a destruction and confusion. Not all people understand CI and P-value very well. The numbers should be explained in detail for anybody to understand what is really happening.

The FGm/C especially in Tanzania is performed by traditional birth attendant and traditional circumciser because they share the same social norm with community they are serving. So whatever punishment and policy is applied, so what is the reason behind? Is the traditional way of research and method can really identify the factors and determinants?

Most of the time reports related to FGM focus on the maternal health related consequences of FGM what about the violation of human right, mental health aspect, female sexual function (it is a deep rooted problem for couples to divorce), infections and pains. Female has a right to enjoy her sexual life but the reality is different in a community which forces female to undergo this practice.

I understood that the questioners are massive for DHS participants and self-administered, in Africa the literacy level is low to understand such kind of questions. And also, most of the time priority will be given for other indicators than such cultural but serious problems. Most indicators have been addressed by interview and supported by laboratory and physical examinations, when we come to the FGM/C case only by interview but at least it has to be triangulated with qualitative as the cases require a deep understanding by different approaches. The factors and determinants have not stated well or not clear, it has to be stated like among the determinants …..Factor is significantly associated with FGM/C or the like than numbers. Numbers can be summarized in table.

The statements appropriate for result have been written in the discussion part, it will be better if it included under result.

N.B The paper lacks consistency, to be clearer. Nothing is said about the following issue under the result part but explained well under discussion. **I think the researcher is not clear about what is the difference between result and Discussion. Some statements like a recommendation.**

“There is a shared belief that the presence of the clitoris made women have a greater desire for sex, predisposing them to promiscuity and marital infidelity[9]”.

Parents with such a belief will look for the service and practice it under strict secrecy[8] **so**…. It is reported that majority of FGM/C is carried to girls aged between infancy and age 15[10]. These are minors who depend entirely to their parents for protection. Studies have shown that community sensitization and education and do better in eradicating FGM/C[8].

Communities and tribal leaders need to understand the consequences of FGM/C and make informed choice. **why???** It is evidenced that there is no relationship between FGM/C and promiscuity and marital infidelity[11]. But rather the FGM/C is reported to reduce the quality of sexual act such as sexual pain, orgasm and sexual desire problems at sexual arousal and difficulties in lubrication[12,13]. It is also responsible for mental illnesses such as depression, somatization and anxiety, Post Traumatic Stress Disorder (PTSD) and sleep disorder[13].

. 4. **Conclusions are presented in an appropriate fashion and are supported by the data.**

Not at all

**5. The article is presented in an intelligible fashion and is written in standard English**.

Not at all

**6. The research meets all applicable standards for the ethics of experimentation and research integrity.**

Yes
7. **The article adheres to appropriate reporting guidelines and community standards for data availability.**

Not at all
