# Supplementary material for: Prevalence and factors associated with female genital mutilation/cutting among Tanzanian women who gave birth in the five years prior to the survey: A population-based study
Source: PLoS One. 2024 Dec 30;19(12):e0310337. doi: 10.1371/journal.pone.0310337 (PMC11684563; doi:10.1371/journal.pone.0310337)
Supplement: Attachment — Submitted filename: Response to Reviewers Comments.docx [file pone.0310337.s003.docx]

**Response to Reviewers’ Comments**

Reviewer #1:

**Comment 1:** Title: it is confusing (somewhere it is determinant and somewhere it is prevalence), but in my reading I understood as it is a prevalence study, that's why you used a cross sectional study design, so please make your title as " prevalence and associated factors of FGM/C..."

**Response**: Yes, the study used a cross-sectional design but it went further by controlling for possible confounders in regression analysis. Inferential statistics are used for analytical studies. Therefore, the study present both descriptive (prevalence) and analytical (determinants) findings.

**Comment 2:** Introduction section: the paragraphs need rearrangement, please keep the natural flow of ideas and your justification is not well stated, that is your interest of this study is not clear, why do you interested to conduct this study?

**Response:** The section is strengthened paragraph one and four

**Comment 3:** Your discussion is not presented in the intelligible way, please rewrite again

**Response:** The section is revised

**Comment 4**: Your recommendation also poor, please write your recommendation based on your findings

**Response:** The recommendations are revised

Comment 5: You said "the study is without limitations......." but you put some limitations, what does that mean?

**Response:** The language is revised; the author’s meaning was the study has limitation

**Reviewer #2:**

Thank you, dear editors, for you’re considering me to review this paper!

It is an interesting topic. However, I have the following comments to the authors for improvement:

**Comment 1:** Abstract:

Method: Do you appreciate the difference between a multivariate and a multivariable logistic regression? In the abstract section, you wrote multivariate logistic regression. Is multivariate logistic regression appropriate for your analysis?

**Response:** The correction is made the study used multivariable logistics regression

Result: it is better to add the CI to AOR

**Response:** Confidence intervals were added

**Comment 2:** Introduction

-It is good to refer to PLOS One as a guideline for reference citation. Your citation is superscripted.

**Response:** The intext citation are revised to adhere to PLOS One instruction to authors

Methods: you state that, ‘The study design was an analytical cross-sectional study design using secondary data and The Tanzania Demographic and Health Survey (DHS) and Malaria

The Indicators Survey (MIS) 2015–2016 dataset was used’.

**Response:** The study was analytical because the analysis went further to controlling for confounders in the analysis. The regression analysis was used to assess the determinants of FGM/C

-Why is pre-test? Since you used secondary data, why were sixteen (16) field teams used for data collection? Why Supervisor? Why interviewers?

**Response:** The explanation in this section is according to the procedure taken by DHS Measure to ensure collection of credible and representative data, the section is revised to carry this meaning

-I expected the data to be extracted from the Tanzania Demographic and Health Survey (DHS) and Malaria Indicators Survey (MIS) 2015-2016 dataset as secondary data. But your data collection procedure describes how you collected primary data? Please try to describe the method clearly.

**Response:** The data were extracted from DHS-MIS, the procedure presented in the manuscript is according to what was done in the acquiring these secondary data

- Sample size calculation and sampling procedures are not clearly stated.

**Response:** The sample size based on the study criteria, women of reproductive age who responded to the question on whether they have undergone FGM/C were extracted from the total sample size and cleaning was done to omit all with missed data. The section is strengthened

- NB: A more detailed method is needed to show how you extract your manuscript data from Tanzania Demographic and Health Survey (DHS) and Malaria Indicators Survey (MIS) 2015-2016 dataset.

**Response:** The section is revised

**Comment 3:** Result:

- What is the importance of the relationship between the characteristics of women of reproductive Age and female genital Mutilation/Cutting Status? You have addressed the associated factors.

**Response:** Relationship is the output of a cross tabulation analysis. It is a descriptive analysis which only describe the existence of a relationship between the independent variable and a dependent variable

- How candidate variables selected for logistic regression analysis from a huge dataset?

**Response:** All variables which showed a significant relationship in the cross-tabulation analysis were selected for logistic regression

-What are the reasons for the wide CI in Zones of Northern (28.41-296.546), Central (67.093- 689.423), Southern Highland (3.49 - 41.298), Lake (4.338 - 44.714) and Eastern (7.299- 80.017) in logistic regression model?

**Response:** A discussion to explain a possible reason for a wide confidence interval is added

**Reviewer #3:** -

Comment 1: There should be a clear objective for research

- What makes this research analytical? it is purely descriptive. if it is analytical cross sectional where is the hypothesis?

- There should be a clear methodology

- Should understand each components of research. the difference between result and discussion

**Response:**

**Comment 2:** It seems a review as it has critically evaluated the available data. It will help for a quick reference otherwise; the full version of the DHS has been available online for anybody.

**Response**: It is true the DHS has a report but the report available is only descriptive. It reports about the prevalence of FGM/C. This study went further by conducting inferential analysis and report the factors associated with FGM/C. The findings from this study explain the characteristics of women of reproductive age who are likely to undergo FGM/C. This information is important for a well-directed effort on eradication the practice.

**Comment 3:** The data has been extracted from the DHS. My concern here is the data has been collected from permanently residents and visitors who stayed for one night, is it really make sense to collect a data from visitors especially on sensitive and culture related topics? Such kinds of practices are done secretly, how visitors will have the information?

**Response:** The individual file was used to extract data which was used in this study. Regarding to the sampling technique used by DHS-Measure, two clusters sampling techniques were used to sample the respondents, the first cluster sampling is selection of villages then the second cluster sampling was the selection of households, all women of reproductive age in the selected household were interviewed on the issue of female genital cutting. The data is a country representative data, the chance that the visitors may have diluted the picture of the FGM/C in Tanzania is minimal.

**Comment 4:** The other concern is In Tanzania the Government is working a lot to create awareness on FGM/C which has been a cause for the dropping of FGM/C from 10% (DHS 2015-16) to 8% (DHS 2022) as it has been justified in the report, there is a discrepancy between the main report and this research. The other point is this is purely cultural issue not related to knowledge and religion not only for Tanzania but also all over Africa. So, such kinds of sensitive issue it will be difficult to change the context only be awareness creation and the approach for the research should be different as it has to use behavioral and norm related model and theories to know the unwritten and unspoken hidden norms related with FGM/C, this will help all concerned bodies to critically plan to challenge the barriers. I do recommend PLOS ONE to critically evaluate papers for a real and meaningful contribution in research world, not for publication purpose but it has to be used as an input for the decision makers and other stake holders.

**Response:** I do agree the practice of FGM/C is decreasing in the country due to the governmental and none governmental efforts. The current study did not study the trend in FGM/C but rather the determinants of FGM/C. The central focus of the current study is to understand the characteristics of women who undergo FGM/C so that to direct the efforts of the Government and non-governmental organizations. The magnitude reported in this study could have differed with the one reported in the DHS report because the current study included only women of reproductive age while the report included all women.

**Comment 5:** From my knowledge is concerned, in Africa the problem is no one is interested to explore a new knowledge but duplication. PLOSE ONE is a appreciated journal which challenges researchers than others, so avoiding such kinds of researches have to be avoided. There are lots of issues should be answered or little is known, the answer of this research topic is known “we have to avoid duplication”.

**Response:** I do agree with the reviewer that PLOSE ONE is a reputable journal and publish solid findings. It is with the same reason I chose to publish with them. The intention of this work is not to duplicate the available information but rather to add to what is already known. The DHS data reported the magnitude of FGM/C in the country and this study went further by reporting the characteristics of women who are more likely to be mutilated.

**Comment 6:** The most recent DHS result has been published; it is enough for reference and input purpose. Policy makers and other concerned bodies can refer the officially launched DHS report. For such kinds of culturally sensitive areas, we should use another innovative method to understand the deep-rooted causes to make a difference. For the publication purpose there are other journals open for any kinds of papers.

**Response:** Findings from this study have additional information which can be used by policy makers to direct their decision making towards the practice

**Comment 7:** The result part is the main part of the paper which peoples quickly refer to understand the context but, in this paper, it has been written mainly in numbers (not interpreted in words) which can cause the readers a destruction and confusion. Not all people understand CI and P-value very well. The numbers should be explained in detail for anybody to understand what is really happening.

**Response:** The numbers are interpreted in the discussion section

**Comment 8.** The FGM/C especially in Tanzania is performed by traditional birth attendant and traditional circumciser because they share the same social norm with community they are serving. So whatever punishment and policy is applied, so what is the reason behind? Is the traditional way of research and method can really identify the factors and determinants?

**Response:** FGM/C has impact in birth outcome. It is the role of the government to make the community aware of that. Punishment without sensitization and raising awareness is not correct. Punishment are used as a negative reinforcement of unwanted behavior. The study identified women’s characteristics which are likely to favor FGM/C

**Comment 9:** Most of the time reports related to FGM focus on the maternal health related consequences of FGM what about the violation of human right, mental health aspect, female sexual function (it is a deep-rooted problem for couples to divorce), infections and pains. Female has a right to enjoy her sexual life but the reality is different in a community which forces female to undergo this practice. I understood that the questioners are massive for DHS participants and self-administered, in Africa the literacy level is low to understand such kind of questions. And also, most of the time priority will be given for other indicators than such cultural but serious problems. Most indicators have been addressed by interview and supported by laboratory and physical examinations, when we come to the FGM/C case only by interview but at least it has to be triangulated with qualitative as the cases require a deep understanding by different approaches. The factors and determinants have not stated well or not clear, it has to be stated like among the determinants …. Factor is significantly associated with FGM/C or the like than numbers. Numbers can be summarized in table.

**Response:** The impact of FGM/C is expanded in the background information

**Comment 10.** N.B The paper lacks consistency, to be clearer. Nothing is said about the following issue under the result part but explained well under discussion. **I think the researcher is not clear about what is the difference between result and Discussion. Some statements like a recommendation.**

**Response:** According to the instruction to authors, results present the output of analysis and the discussion serves the purpose of interpretation and discussion
