# Supplementary material for: Prevalence and factors associated with female genital mutilation/cutting among Tanzanian women who gave birth in the five years prior to the survey: A population-based study
Source: PLoS One. 2024 Dec 30;19(12):e0310337. doi: 10.1371/journal.pone.0310337 (PMC11684563; doi:10.1371/journal.pone.0310337)
Supplement: Attachment — Submitted filename: Point to point response to reviewers.docx [file pone.0310337.s004.docx]

**Point to point response to reviewers’ Comments**

**General comment**

This is an interesting paper that uses survey data to assess the prevalence of FGM and factors associate with the FGM. The paper continues to add to the body of knowledge on FGM. Although the paper provides useful information on the factors that are associated with FGM in Tanzania, the structure and the presentation in the manuscript may need some copy editing and formatting to ensure flow before publication. Detailed areas of recommendations are provided below

**Response:** Thank you

**Comment 1.**

Title: The title is well written providing information on the population and the study setting as well as the design employed. I however believe this could still be shortened as it seems too long. Alternative descriptive words could be used in the main body of the manuscript. Tanzania is repeated twice in the e title and could be omitted to shorten the title. Second, is the use of the term ‘determinants. This implies that the study is showing a causal relationship when in essence the study’s focus is to assess the factors associated with FGM.

**Response:** The title is shortened and the term determinants is replaced with factors

**Comment 2**

Abstract: The abstract is well structured and concise. The word determinant and factors associated are used interchangeably in methods section when in deed they are distinct

**Response:** The term determinants is replaced by factors throughout the document

**Comment 3**

Introduction: Generally, the introduction section is ok and provides the context of the study as well as the justification. There are a few areas that may need to be refined

a. for example, the sentence that begin with, “The procedure is performed using unsterilized tools…..” seems to generalize that all FGMs and provides a biased analysis of the problem.

**Response:** The sentence is re-written and the bias in the sentence is addressed- line 87-90

b. The statement that begins with “improperly performed…” imply that there are proper ways of performing FGM.

**Response:** The sentence is re-written, focus is on surgical incision performed locally with untrained health care providers- line 92-98

c. There is a missing ‘to’ after according in the third paragraph.

**Response:** The correction is made -113

d. The word female genital mutilation/cutting has been abbreviated as FGM and can use the abbreviation thereafter. Otherwise the authors keep going back and forth with the use of the abbreviation and full description in the body of the manuscript.

**Response:** The correction is made throughout the document

e. Any reference to the high prevalence rates in the areas listed in Tanzania?

**Response:** A paragraph is added which describe the regional disparities in FGM/C in Tanzania-line 107-112

Comment 4

Methods:

a. It would be helpful to provide a description of the TDHS-MIS and then the methods for this study. As it is, the methods being described in this study belongs to the TDHS-MIS. How was the data collected/abstracted for this study?

**Response:** The section has been revised in lines 171-185 to provide a clearer description of the method used in acquiring the DHS dataset..

b. What data cleaning procedures were conducted?

**Response:** The cleaning procedure has been revised for clarity, as detailed in lines 193-199.

c. How was missed data managed/handled? The data from women who were missing the information on whether or not they underwent FGM could bias your results – I can imagine the social desirability bias could have affected your findings here.

**Response:** The proportion of missing data is now included in line 198. Respondents with missing data were excluded from the analysis. The authors acknowledge the reviewer's observation that this exclusion may have introduced bias into the findings, and this potential bias is addressed as a limitation in the study-line 444-448

d. Why was the age categorized? For women aged less than 20 years, what was the lower age limit. Similarly, what was the upper age limit for those who were 34 and above?

**Response:** The objective was to analyze disparities in the prevalence of FGM/C across three distinct age categories of women of reproductive age: teenage mothers (15-19 years), middle-aged mothers (20-34 years), and older mothers (35-49 years). The age range for the third category has been revised to 35-49 years.

e. I want to believe the survey collects data from all women, and if this is not correct then your study background or the survey in which your study is based on, needs to be well described.

**Response:** The survey collected data exclusively from women of reproductive age. The aim of the current study has been modified in lines 144-149 to better reflect its focus on understanding the prevalence and contributing factors of FGM/C within this population.

f. In the dependent variable section, delete the sentence that starts with ...Literature…

**Response:** The sentence is deleted- line 236-239

g. Which statistical analysis software was used to conduct the analysis?

**Response:** The statistical analysis software used has been added to the Data Analysis section, specifically in line 246.

Comment 5

Results:

a. The results on circumcision is confusing. Were participants asked a follow up question on whether the flesh was removed or not? If that is the case, this is not provided in the methods section to understand this finding. Additionally, the finding with about 9% of the participants reporting not knowing whether or not the flesh was removed is more confusing – how did these persons know that they underwent FGM/C? Was there an examination of the genitalia to determine the responses?

**Response:** The methods section has been enhanced, with a more detailed explanation of the dependent variable –line 236-243. However, it should be noted that the reliance on self-reported data may introduce bias into the findings. This potential limitation is addressed in the study's limitations section-

b. Some figures could be left out as a sentence in a text communicated the information effectively, otherwise the figure just repeats what is in the text. If inserting figure 4, then there is no need of explaining everything in that figure within the text.

**Response:** Agreed, figure 2 and 3 are removed

Comment 6

Discussion: secretly

a. This section is a little hard to follow. For example, paragraph 2is a little loaded with a lot of information that is hard to make out the implication of the study’s findings in comparison with what has been published out there.

**Response.** The rearrangement of information is done between paragraph 1 and 2- line 330-337

b. Paragraph 3 of the discussion does not discuss any of the study findings.

**Response:** An introductory sentence is added to connect with previous presented results

c. Paragraph 5 of the discussion does not seem to have provided a probable justification for the wide CI in the findings.

**Response:** The justification is clarifies-line 382-483

d. What could have explained the study finding that education levels and rural dwelling were not significantly associated with FGM? Yet other studies have documented so?
e. Response: A possible explanation is added -line 427-430

Conclusion: The statement in the conclusion on the next step based on the findings should be focused. It is too general to relate to the findings of the study. What can be done about the women who have already undergone FGM and want this act to continue, hence the likely of them engaging this to their young girls?

**Response:** The section is re-written
